# Sclerostin Promotes Bone Remodeling in the Process of Tooth Movement

**DOI:** 10.1371/journal.pone.0167312

**Published:** 2017-01-12

**Authors:** Rui Shu, Ding Bai, Tzongjen Sheu, Yao He, Xianrui Yang, Chaoran Xue, Yiruo He, Mengyuan Zhao, Xianglong Han

**Affiliations:** 1 Department of Orthodontics, State Key Laboratory of Oral Disease, West China School of Stomatology, Sichuan University, Chengdu, Sichuan, China; 2 School of Medicine and Dentistry, University of Rochester Medical Center, Rochester, New York, United States of America; Universite de Nantes, FRANCE

## Abstract

Tooth movement is a biological process of bone remodeling induced by mechanical force. Sclerostin secreted by osteocytes is mechanosensory and important in bone remodeling. However, little is known regarding the role of sclerostin in tooth movement. In this study, models of experimental tooth movement were established in rats and mice. Sclerostin expression was investigated with immunohistochemistry staining, and osteoclastic activity was analyzed with tartrate-resistant acid phosphatase (TRAP) staining. MLO-Y4 osteocyte-like cells underwent uniaxial compression and tension stress or were cultured in hypoxia conditions. Expression of sclerostin was assessed by RT-qPCR and ELISA. MLO-Y4 cells were cultured with recombinant human sclerostin (rhSCL) interference and then co-cultured with RAW264.7 osteoclast precursor cells. Expressions of RANKL and OPG were analyzed by RT-qPCR, and osteoclastic activity was assessed by TRAP staining. During tooth movement, sclerostin was expressed differently in compression and tension sites. In SOST knock-out mice, there were significantly fewer TRAP-positive cells than in WT mice during tooth movement in compression sites. In-vitro studies showed that the expression of sclerostin in MLO-Y4 osteocyte-like cells was not different under a uniaxial compression and tension force, whereas hypoxia conditions significantly increased sclerostin expression in MLO-Y4 cells. rhSCL interference increased the expression of RANKL and the RANKL/OPG ratio in MLO-Y4 cells and the osteoclastic induction ability of MLO-Y4 cells in experimental osteocyte-osteoclast co-culture. These data suggest that sclerostin plays an important role in the bone remodeling of tooth movement.

## Introduction

The biological mechanism of orthodontic tooth movement is bone remodeling induced by mechanical force. This process involves bone resorption in compression sites and bone formation in tension sites [1–3]. Previous studies on orthodontic tooth movement have revealed the important function of osteoblasts and osteoclasts [4–6], and some studies have investigated the roles of osteocytes and their secreted cytokines in orthodontic tooth movement.

Osteocytes, the major cellular components of mature bone, are the main mechanosensory cells in bone tissue [7]. Osteocytes have been found to act as orchestrators of bone remodeling through the regulation of both osteoclastic and osteoblastic activity [8–11]. In addition, osteocytes function as endocrine cells and regulate bone metabolism [12–16]. Matsumoto [17] found that with ablation of osteocytes in the alveolar bone, the tooth movement distance was significantly decreased, and the number of active osteoclasts in the compression site was significantly reduced. These findings indicated the important role of osteocytes in tooth movement. However, the mechanism by which osteocytes regulate the function of osteoclasts and bone remodeling during tooth movement remains unknown.

Sclerostin, which is encoded by the SOST gene and predominantly secreted by mature osteocytes, was discovered to be an antagonist of the canonical WNT pathway [18–20]. By competitively binding to the WNT co-receptor, LRP5/6, Sclerostin induces phosphorylation and degradation of β-Catenin and hinders the activation of osteoblasts. An SOST gene mutation in humans causes Van Buchem disease or sclerosteosis, which are both characterized as hyperostosis [21, 22]. A targeted deletion of the SOST gene in mice increased bone formation and improved the healing of bone defects [23, 24]. The inhibitory effect of sclerostin on osteoblasts has been well illustrated, whereas only a few studies focus on the relationship between sclerostin and osteoclasts. Wijenayaka found that sclerostin increased the RANKL expression of MLO-Y4 osteocyte-like cells [25]. Ota’s study showed that sclerostin was expressed in osteoclasts from aged mice and reduced the osteoclast-stimulated mineralization [26]. Therefore, the underlying mechanism of sclerostin- influenced osteoclastogenesis warrants additional research.

The expression of sclerostin by osteocytes is regulated by mechanical loading. The expression of sclerostin is reduced when mechanical loading is increased and increased when mechanical loading is decreased [27–29]. The mechanosensory characteristics of sclerostin indicate that it is a key protein in bone remodeling under mechanical stimulation.

Orthodontic tooth movement is a biological process induced by mechanical loading. We researched the participation in bone remodeling by sclerostin expression of alveolar osteocytes during tooth movement. The study aims were to identify 1) the pattern of sclerostin expression in alveolar bone during tooth movement; and 2) the effects and the underlying mechanisms of sclerostin on tooth movement bone remodeling.

## Materials and Methods

The experiment protocol was approved by the Animal Care and Ethics Committee of West China School of Stomatology, Sichuan University (NO: SKLODLL2012A025).

### Animals and experimental tooth movement models

The rat experimental tooth movement procedures were as follows: 35 male Spraque-Dawley rats, 6- to 8-week-old, with a weight range between 180 g to 220 g were obtained from the experimental animal center of Sichuan University. The methods used were as previously described [30]. In brief, rats were anesthetized by an intraperitoneal injection of 10% chloral hydrate (3 mg/kg). A mouth-prop made from a 0.036-in stainless steel wire was used to maintain the mouth in an open position and expose the first maxillary molars. A 0.010-in stainless ligature was threaded beneath the contact point between the first and second molars. A low force nickel-titanium coil spring (3M Unitek, Monrovia, CA) was tightly ligated to the first molar with the 0.010-in stainless steel ligature. The other end of the coil spring was attached to the incisors with a 0.010-in stainless steel ligature, and the force of the coil spring was calibrated to 20 g with a dynamometer. In addition, 0.5 mm-deep grooves were prepared on the labial, mesial and distal surface of the incisors to prevent dislodging of the attached ligatures. The ligatures were fixed to the incisors with light-cured resin after acid-etching of the enamel. The coil springs were checked every day, and light-cured resin would be added if necessary. Five rats were separately and randomly euthanized at day 1, 3, 5, 7, 14, 21 and 28 and underwent further analysis. Immunohistochemistry staining was used to analyze the expression of sclerostin. Tartrate-resistant acid phosphatase (TRAP) staining was used to measure the osteoclastogenesis activity.

Tooth movement procedures in the experimental mice were as follows: twenty-five 8-week-old C57BL/6 mice (obtained from the experimental animal center of Sichuan University) and twenty-five complete SOST KO mice (kindly supplied by Professor Jian Q Feng from Baylor College of Dentistry) underwent experimental tooth movement. Five mice in each group were randomly selected for euthanization by CO_2_ inhalation followed by cervical dislocation at days 2, 4, 6, 8, and 10, and the maxilla was harvested and underwent further analysis.

The mice experimental tooth movement procedure was similar to that in rats. The mice underwent general anesthesia by abdominal injection of 0.08 milliliters of 10% chloral hydrate. A mouth-prop was used in each mouse to keep the mouth open. A super-elastic nickel titanium coil spring was activated by being ligated between the left maxillary first molar and the incisor. The tooth movement force was calibrated to 0.05 N (5.1g on dynamometer), and a stainless ligature was fixed to the prepared groove on the incisors with light-cured resin after acid etching of the enamel. The coil springs were checked every day, and light-cured resin would be added if necessary.

The rats and mice were kept warm under incandescent light until they were fully awake, and each group of five mice was housed in a single cage after the procedures. The mice were maintained on a light-dark cycle (12 h light, 12 h dark with lights on at 8:00 AM) at room temperature of 23 ± 1°C and provided standard food and water ad libitum before the experiments. All of the rats and mice were cared for in accordance with international standards for animal welfare compliant with the guidelines of the Animal Research Committee of the West China School of Stomatology, Sichuan University.

### Cell culture

The murine origin MLO-Y4 osteocyte-like cells were kindly provided by Dr. Lynda Bonewald (University of Missouri-Kansas City, Kansas City, MO). The murine origin RAW264.7 osteoclast precursor cells were purchased from ATCC. MLO-Y4 was cultured in α-MEM medium supplemented with 5% fetal bovine serum, 5% calf serum, and 1% penicillin and streptomycin (PS). RAW264.7 was cultured in a DMEM medium (GIBCO) supplemented with 10% FBS (GIBCO) and 1% PS (GIBCO). The cell lines were cultured in a humidified incubator at 37°C and 5% CO_2_.

The MLO-Y4 cells were separately subjected to cyclic uniaxial compression and tension stress (magnitude, 4000 μ strains; frequency, 0.5 Hz) for 1 h, 3 h, 6 h, 12 h and 24 h. The stress was supplied with a uniaxial 4-point bending system (developed at Sichuan University). The SOST mRNA levels were analyzed byRT-qPCR.

The hypoxia culture methods were as previously described [31, 32]. The MLO-Y4 cells were separately cultured in normoxic- (20% O_2_) and hypoxic- (1% O_2_) condition incubators with 5% CO_2_ and balanced N_2_ for 48 h. The SOST mRNA level was analyzed byRT-qPCR, and the sclerostin protein level was analyzed by enzyme-linked immunosorbent assays (ELISA).

The MLO-Y4 cells were seeded into type I collagen (Corning) 6-well plates and cultured until the cells achieved 80% confluency. The MLO-Y4 cells were exposed to (10 ng/ml, 30 ng/ml, 50 ng/ml, and 100 ng/ml) recombinant human sclerostin (rhSCL, R&D Systems, MN, USA) for 5 days. The RANKL and OPG mRNA levels were analyzed by RT-qPCR. The protein level of RANKL was analyzed by ELISA assays.

The co-culture methods of MLO-Y4 and RAW264.7 were as previously described [10]. The MLO-Y4 cells were seeded at a density of 500 cells/cm^2^ in collagen-coated 24-well plates (day 0). The RAW264.7 cells were added at a density of 2500 cells/cm^2^ at day 2 and then co-cultured in DMEM medium supplemented with 10% FBS and 1% PS for 7 days. The cells were fixed, and the osteoclastogenesis was analyzed with (TRAP) using a leukocyte acid phosphatase kit (Sigma, USA) as instructed in the product datasheet. The TRAP-positive cells were counted under light microscopy at 20X magnification by 2 double-blinded investigators. Each well was counted three times. The average number of TRAP-positive cells was compared between the groups with or without the rhSCL treatment.

### Histology, immunohistochemistry, and TRAP staining

Harvested rat and mouse maxillary tissues were fixed, decalcified (rats for 30d, mouse for 20d) and embedded in paraffin. Section were cut in 4μm and used for immunohistochemistry (IHC) and TRAP staining. The periodontal ligament (PDL) tissue and alveolar bone mesial to the upper one-third of the mesial root was identified as the compression sites and the PDL and alveolar bone distal to it was identified as the tension sites. IHC staining was done with SOST polyclonal antibody (1:400 diluted in PBS. R&D systems, MN, USA) and counterstained with Hematoxylin. The images were captured under light microscope at the same setting. The percentage of sclerostin positive cells over the total cells in compression and tension area was measured by Image-Pro Plus (5.0, Media Cybernetics, Bethesda, Md). TRAP staining was done with a leukocyte acid phosphatase kit (Sigma, USA) as instructed in the product datasheet. The TRAP positive multinuleated cells in the bone surface compression and tension area were quantified as osteoclast numbers per bone surface (mm^-^) with Image-Pro Plus software.

### Preparation of RNA and RT-qPCR

Total RNA was extracted from MLO-Y4 cells after rhSCL interference with Trizol reagent (Invitrogen, Carlsbad, Calif). Complementary DNA (cDNA) was prepared by using a TaKaRa PrimerScript 1^st^ Strand cDNA Synthesis Kit (TaKaRa, Tokyo, Japan) andRT-qPCR was done as previously described [33]. Briefly, a total 20μL cDNA mixture was subjected to PCR amplification. The amplification of glyceraldehyde-3-phosphate dehydrogenase (GAPDH) was used as an internal control. The primer sets were shown in Table 1.

**Table 1:** Primer sequences of Real-time RT-qPCR.

GAPDH	Forward	5’-AGGTCGGTGTGAACGGATTTG-3’
	Reverse	5’-GGGGTCGTTGATGGCAACA-3’
SOST	Forward	5’ -AGCCTTCAGGAATGATGCCAC-3’
	Reverse	5’-CTTTGGCGTCATAGGGATGGT-3’
RANKL	Forward	5’-CGCTCTGTTCCTGTACTTTCG-3’
	Reverse	5’-GAGTCCTGCAAATCTGCGTT-3’
OPG	Forward	5’-CCTTGCCCTGACCACTCTTAT-3’
	reverse	5’- CACACACTCGGTTGTGGGT-3’

### Enzyme-linked immunosorbent assay (ELISA)

The culture medium of MLO-Y4 was collected for ELISA. The protein level of sclerostin and RANKL released into the culture supernatants were measured with mouse Sclerostin ELISA kit (Boster, Wuhan, China) and mouse RANKL ELISA kit (Abcam, USA), according to product’s datasheet.

### Statistical analysis

The statistical analysis was performed with SPSS 17.0. All of the results were presented as the mean and the standard deviation and assessed by the independent student’s *t*-test or one-way ANOVA analysis, and turkey post-hoc tests were performed. A *P*<0.05 was considered to be statistically significant.

## Results

### Expression of sclerostin was different between the compression and tension sites in response to tooth movement in rats

**Fig 1:**
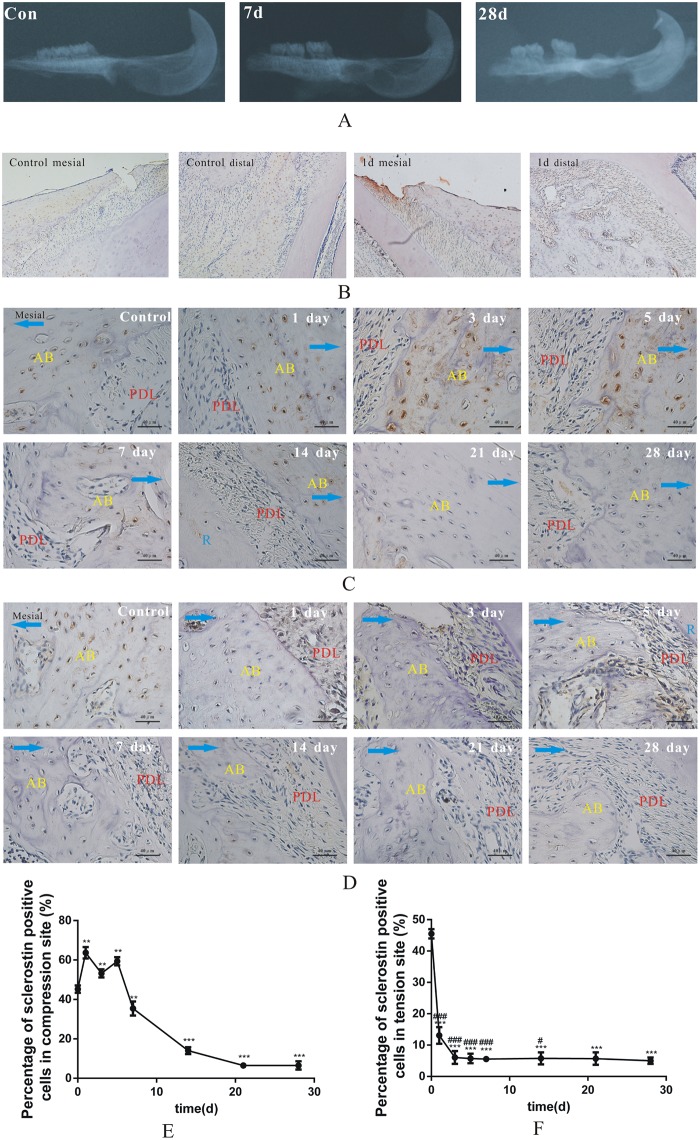
Expression of sclerostin was different between the compression and tension sites during tooth movement. (A) X-ray images showed the mesial movement of the first molars at 0 d, 7 d and 28 d. (C) IHC of sclerostin at the compression sites. Sclerostin maintained high expression at 1d, 3d, 5d and 7d and gradually diminished until 28 d. Alveolar bone (AB), PDL (periodontal ligament), and R (root). The blue arrow indicated the mesial side in control group and the direction of tooth movement in experiment groups. (D) IHC of sclerostin at the tension site. Sclerostin immediately decreased and was continually maintained at the low level during 28 days of tooth movement. AB (alveolar bone), PDL (periodontal ligament), R (root). (E) Semi-quantification of IHC of sclerostin at the compression sites. n = 3,**p<0.01 vs control, *** p<0.001 vs control. (F) Semi-quantification of IHC of sclerostin at the tension sites. n = 3, ***p<0.001 vs control, ^#^p<0.05 vs compression site, ^##^p<0.01 vs compression site.

To address the expression of sclerostin in alveolar bone during tooth movement, we established an experimental tooth movement model in rats. In the X-ray images of the harvested samples, the distance of the first molars’ mesial movement increased with time, and there was almost 1 mm between the first and second molars at d 28 of tooth movement (Fig 1A). The IHC staining showed that at the control site, sclerostin was broadly localized in the alveolar bone, and there was no difference between the mesial and distal sites (Fig 1B and 1C). In the tooth movement group, sclerostin expression remained high at the compression sites and was significantly reduced at the tension sites 1 day after tooth movement force loading (Fig 1B). To address the changes of sclerostin expression during tooth movement, we traced the protein level of sclerostin at 1 d, 3 d, 5 d, 7 d, 14 d, 21 d, and 28 d after tooth movement loading (Fig 1C and 1D). At the tension site, the expression of sclerostin decreased immediately at 1 d and continually maintained the low level through the 28 days of tooth movement (Fig 1F). At the compression sites, the expression of sclerostin slightly increased and remained at a high level in the first week after tooth movement force loading. After 7 days, the sclerostin level gradually diminished until 28 d (Fig 1E). These results indicated that compression and tension might have different effects on the sclerostin expression of osteocytes.

### The sclerostin expression of MLO-Y4 cells was increased under hypoxia conditions, whereas it is not different under uniaxial compression and tension stress

**Fig 2:**
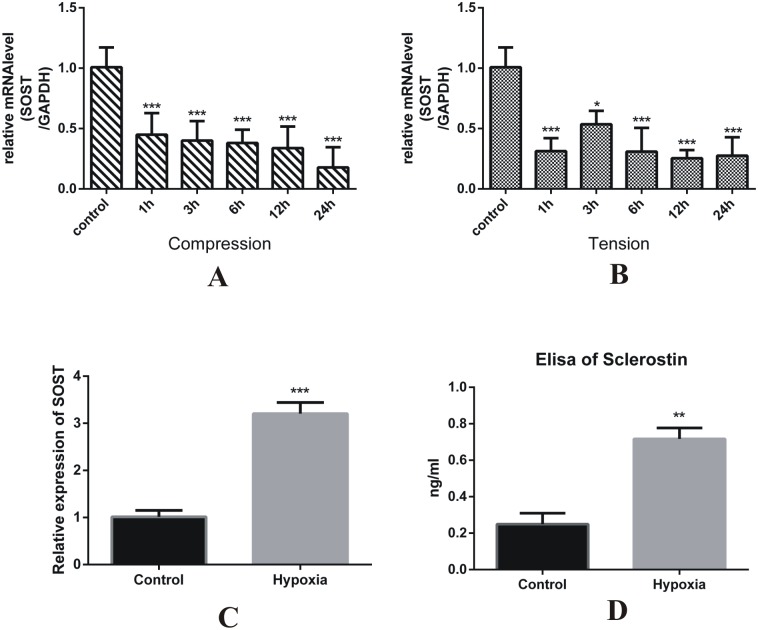
The sclerostin expression of the MLO-Y4 cells was increased under hypoxic conditions, whereas it was not different under uniaxial compression and tension stress. (A) The relative mRNA expression of SOST of the MLO-Y4 cells was decreased under compression stress. ***p<0.001 vs control. (B) The relative mRNA expression of SOST of the MLO-Y4 cells was decreased under compression stress. ***p<0.001 vs control. (C) The relative mRNA expression of the SOST mRNA of the MLO-Y4 cells was increased 48 h after being cultured under hypoxic conditions. ***p<0.001 vs control. (D) The release of sclerostin by the MLO-Y4 cells that was determined using ELISA increased 48 h after being cultured under hypoxic conditions. **p<0.01 vs control.

To further identify whether compression and tension stress have different effects on the sclerostin expression of osteocytes, MLO-Y4 osteocyte-like cells were loaded with uniaxial compression and tension stress. The RT-qPCR results showed that the SOST expression level was immediately reduced 1 h after stress loading and maintained a low level through 24 hours in the compression and tension group. There was no difference between compression and tension groups (Fig 2A and 2B), which indicated that compression and tension stress have identical effects on the sclerostin expression of osteocytes in vitro. We hypothesized that the different expression of sclerostin in compression sites of tooth movement was induced by the hypoxic conditions. To investigate the effect of hypoxia on the expression of sclerostin, the MLO-Y4 cells were cultured in hypoxic conditions; the results showed that the SOST mRNA level was significantly increased compared to that in the normoxic condition (Fig 2C and 2D). Our results indicated that the varying expression of sclerostin in the compression and tension sites during tooth movement might result from different biological microenvironments caused by conditions such as hypoxia and not by the difference between compression and tension.

### The osteoclastic activity was consistent with the sclerostin expression at the compression sites during tooth movement

**Fig 3:**
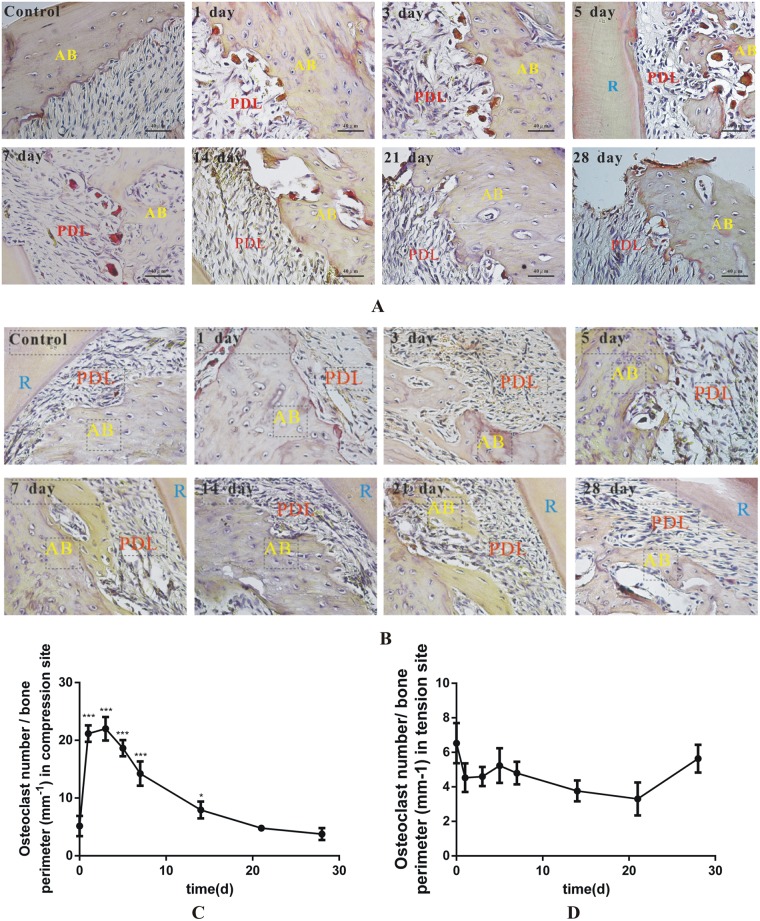
The TRAP staining results showed that the osteoclastic activity was consistent with the sclerostin level at the compression sites during tooth movement. (A) The TRAP-staining results in the compression sites at 0 d, 1 d, 3 d, 5 d, 7 d, 14 d, 21 d and 28 d during tooth movement. The TRAP-positive cells were identified as red multinuclear cells near the alveolar bone. Alveolar bone (AB), PDL (periodontal ligament), R (root). (B) The TRAP-staining results in the tension sites at 0 d, 1 d, 3 d, 5 d, 7 d, 14 d, 21 d and 28 d during tooth movement. The TRAP-positive cells were identified as red multinuclear cells near the alveolar bone. Alveolar bone (AB), PDL (periodontal ligament), R (root). (C) The quantification of TRAP-positive cells per bone surface (mm^-1^) at the compression sites. The number of active osteoclasts increased to the apex in the first three days and gradually decreased with time. *p<0.05 vs 0 d, ***p<0.001 vs 0 d. (D) The quantification of TRAP-positive cells per bone surface (mm^-1^) at the tension sites. The number of active osteoclasts increased to the apex in the first three days and gradually decreased with time.

To address the osteoclastic bone resorption in the compression sites of tooth movement in rats, TRAP staining was used to analyze the osteoclastic activity at the compression sites during tooth movement. In the control group, there were few osteoclasts near the alveolar bone. TRAP- positive osteoclasts per bone surface immediately increased to a high level on day 1 after the usage of the tooth movement appliances. The change of TRAP-positive cells at the compression sites showed the active osteoclasts increased to the apex in the first three days and gradually decreased with time (Fig 3A and 3C). While in tension sites, the TRAP-positive cells per bone surface showed no significant change during the whole experiment (Fig 3C and 3D). After comparing the changes in the number of osteoclasts and sclerostin expression at the compression sites and tension sites during tooth movement, we found that the two had a similar pattern. We hypothesized that there might be a correlation between the sclerostin expression and osteoclastic activity during tooth movement.

### Osteoclastic activity was decreased in the SOST deficient mice compared to the WT mice during tooth movement

**Fig 4:**
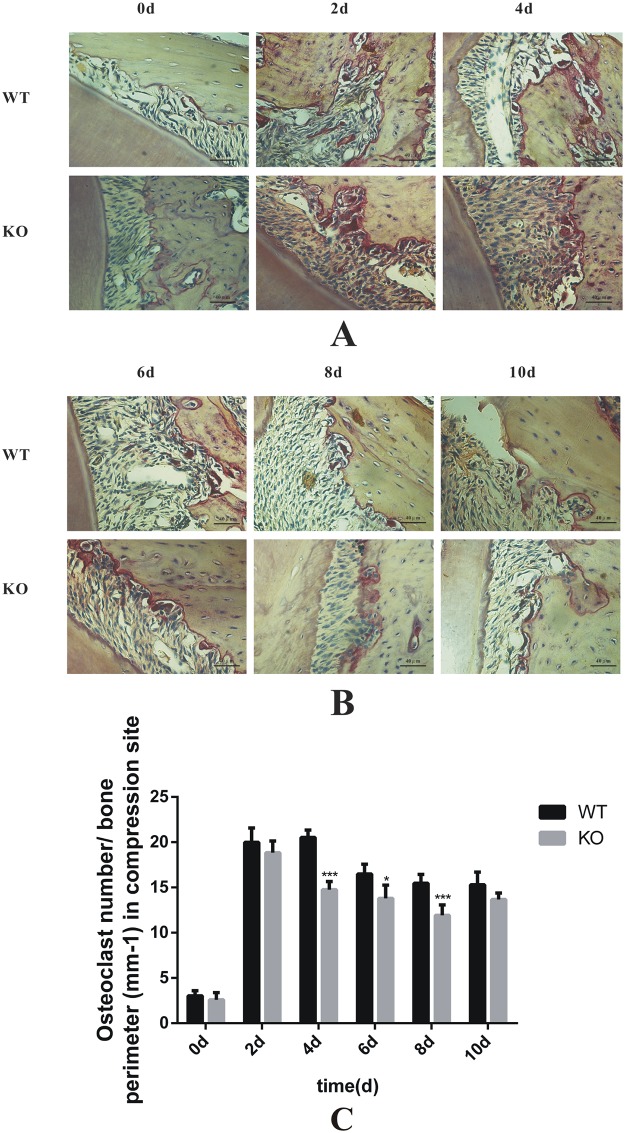
Osteoclastic activity was decreased in the SOST deficient mice compared to the WT mice during tooth movement. (A) TRAP staining at the compression sites in the WT and SOST KO mice at 0 d, 2 d and 4 d. (B) TRAP staining at the compression sites in the WT and SOST KO mice at 6 d, 8 d and 10 d. (C) Quantification of the osteoclasts number per bone surface (mm^-1^) at the compression site of mice tooth movement. The osteoclast number/bone perimeter increased to the apex at day 4 and gradually decreased through day 10 in the SOST KO mice and WT mice. The number of osteoclasts was significantly lower in the SOST KO mice than in the WT mice at 4d, 6d, and 8d. *p<0.05 vs WT. ***p<0.001 vs WT.

To address whether there was a correlation between sclerostin expression and osteoclastic activity at the compression sites, we established a tooth movement model with SOST KO mice and WT C57BL/6 mice. IHC assured that sclerostin expression was totally deleted in SOST KO mice (S1 Fig). TRAP staining showed that the number of osteoclasts increased to the apex at day 4 and gradually decreased through day 10 in the SOST KO mice and WT mice. The statistical analysis demonstrated that there were fewer osteoclasts in the SOST KO mice than in the WT mice (Fig 4A and 4B). The difference was significant at 4d, 6d, 8d and 10d (Fig 4C)). The results supported our hypothesis that there was a relationship between sclerostin and osteoclastic activity at the compression sites during tooth movement. Without sclerostin, the osteoclastic activity decreased at the compression sites during tooth movement.

### Sclerostin enhanced RANKL expression and osteoclastic inducible ability of osteocytes

**Fig 5:**
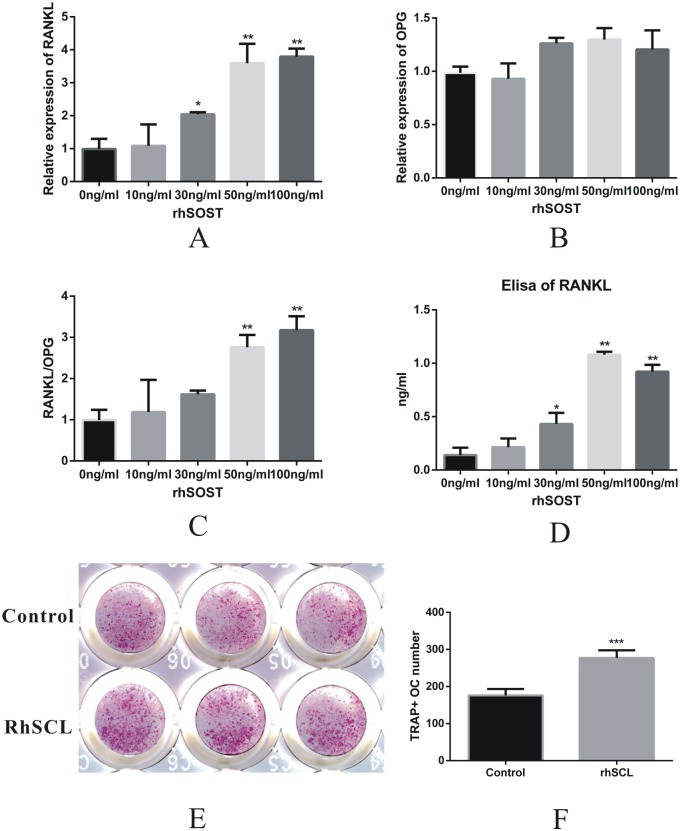
Sclerostin enhanced RANKL expression and osteoclastic inducible ability of osteocytes. (A) Relative mRNA expression of RANKL in the MLO-Y4 cells under 0 ng/ml, 10 ng/ml, 30 ng/ml, 50 ng/ml and 100 ng/ml rhSCL interference. The expression of RANKL was significantly increased with 30 ng/ml, 50 ng/ml and 100 ng/ml. *p<0.05 vs 0 ng/ml. **p<0.01 vs 0 ng/ml. (B) The relative mRNA expression of OPG in the MLO-Y4 cells under rhSCL interference. No significant change in the OPG level. (C) The ratio of RANKL/OPG was significantly increased under 50 ng/ml and 100 ng/ml rhSCL interference. **p<0.01 vs 0 ng/ml. (D) The release of RANKL by MLO-Y4 was determined by ELISA under rhSCL interference. **p<0.01 vs 0 ng/ml. (E) The TRAP staining of the co-culture of the MLO-Y4 and RAW264.7 cells with or without rhSCL interference. (F) There was a significantly greater number of TRAP-positive cells in the rhSCL group than in the control group. ***p<0.001.

In the tooth movement experiment in mice, the RANKL expression was lower at the compression sites in the SOST KO mice than in the WT mice (S2 Fig), which might be the mechanism of the function of sclerostin on osteoclastogenesis. To further study the relationship between sclerostin and osteoclastogenesis. We cultured osteocyte-like cell line MLO-Y4 under rhSCL interference at 10 ng/ml, 30 ng/ml, 50 ng/ml and 100 ng/ml concentrations. We found that sclerostin increased the mRNA expression of RANKL in the MLO-Y4 cells in a dose-dependent way (Fig 5A), whereas the mRNA level of OPG showed no difference under rhSCL interference (Fig 5B). The RANKL/OPG ratio was significantly increased with 50 ng/ml and 100 ng/ml rhSCL interference. Then, we co-cultured MLO-Y4 cells and osteoclast RAW264.7 precursor cells in a medium supplemented with 50 ng/ml rhSCL. TRAP staining was used to demonstrate the number of osteoclasts 7 days after the co-culture. The results showed that the TRAP-positive osteoclasts were increased to a significantly greater degree in the rhSCL-supplemented group than in the group without rhSCL supplementation (Fig 5E and 5F), which indicated that sclerostin enhanced osteocyte-induced osteoclastogenesis.

## Discussion

In this study, we found that sclerostin was directly related to osteoclastic activity in compression sites of tooth movement. First, orthodontic force induced different sclerostin expression patterns between compression and tension sites during tooth movement. Second, TRAP staining showed that the change of osteoclastic activity was similar to that of sclerostin expression in compression sites. Third, mice experimental tooth movement model demonstrated that the osteoclastic activity was decreased in SOST KO mice compared to that in WT mice. Fourth, compression and tension have the identical effect on sclerostin expression, whereas hypoxia conditions, which exist in compression sites during tooth movement, increased the sclerostin expression by osteocytes in in-vitro research. Finally, in-vitro studies showed that rhSCL enhanced the expression of RANKL and the RANKL/OPG ratio in osteocyte-like MLO-Y4 cells. Co-culture of MLO-Y4 and RAW256.7 cells showed that rhSCL interference increased the number of TRAP-positive osteoclasts.

Sclerostin was found to be a critical protein in bone remodeling, and its expression was influenced by mechanical force stimulation [27, 29, 34]. Orthodontic tooth movement was demonstrated to be biological bone remodeling induced by mechanical force. Therefore, explaining sclerostin expression and the role of sclerostin in tooth movement bone remodeling was necessary. Two recently published papers investigated sclerostin expression in tooth movement models [35, 36]; however, the researchers only studied the expression of sclerostin at a single time point. To our knowledge, this study is the first one to trace the expression pattern of sclerostin throughout an entire orthodontic cycle (four weeks) in rats. In this study, the change patterns of sclerostin expression in the compression and tension sites during tooth movement were different. Our result was partially in agreement with a study by Rangiani’ [36], who reported an increase in SOST expression at the compression site and a reduction in expression at the tension site 8 days after orthodontic force loading in a tooth movement model in mice. The different expression pattern of sclerostin in compression and tension sites during tooth movement was a novel finding, which indicated that additional research on the biological function of sclerostin in toothmovement is needed.

In-vitro studies have shown that the effects of uniaxial compression and tension stress on SOST expression in MLO-Y4 cells are the same. Both compression and tension stress decreased the SOST expression. Therefore, the different expression patterns of sclerostin in compression and tension sites during tooth movement might not be induced by the difference between compression and tension force. Researches have shown that many biological changes occur during tooth movement. Hypoxia and ischemia were shown to be responsible for osteoclastic and aseptic inflammation in compression sites during tooth movement [37, 38]. From now on, the results of the research on the effects of hypoxia on sclerostin expression have been controversial. Genetos found that osteoblasts and osteocytes cultured under hypoxia revealed decreased sclerostin transcript and protein [31]. Chen’s study demonstrated that sclerostin was upregulated in osteoblast during hypoxia [32]. To clarify this controversy, we cultured MLO-Y4 cells in hypoxia conditions and found that the transcript and protein levels of sclerostin were upregulated, which was in agreement with Chen’s research. Considering the in-vitro results, we concluded that hypoxia induced by occluded periodontal tissue vessels was one of the reasons for the different expression patterns of sclerostin in compression sites during tooth movement.

In tooth movement experiment, the expression of sclerostin remained low level 28 days after the beginning of tooth movement in both compression and tension site. This could be explained by the vessel changes during tooth movement. Khouw and Goldhaber investigated the vascular changes in periodontal ligaments during tooth movement in rhesus monkeys and German shepherd dogs. They found that the vessels on tension side were widened while that in compression side were completely occluded after 24h and 72h of force application [39]. The occluded vessels in compression site induced the hypoxia. As we know, hypoxia would increase the expression of sclerostin, while stress decrease it. It seemed that hypoxia had stronger effect than stress on expression of sclerostin in compression site at the beginning of tooth movement. This explained the increase of sclerostin in compression site at the 1^st^ week of tooth movement. Khouw and Goldhaber [39] also found increased numbers of new blood vessels near the compression bone resorption areas after seven days of force application. That means 1 week after the beginning of tooth movement, the blood supply was recovered in compression site, and the hypoxia was decreased in this area. This phenomenon explained why the expression of sclerostin in compression site was gradually decreased after 7 days during tooth movement. The tooth movement force still remained even 28 days after the beginning of tooth movement. As the hypoxia diminished with the newly formed blood vessels in the compression site, both compression and tension stress decreased the expression of sclerostin, the mechanical stimulation continuously downregulated the expression of sclerostin in both compression and tension site.

In the rat tooth movement experiment, we found that the changes of osteoclastic activity and sclerostin expression were similar. The tooth movement experiment with SOST KO mice showed that the osteoclastic activity in the compression sites was reduced with the deficiency of sclerostin. These two results indicated that sclerostin had a direct effect on bone resorption during tooth movement. Previous research had clarified that sclerostin had an inhibitory function on osteoblastic activity by antagonizing the canonical WNT pathway; however, few studies have been conducted on the effects of sclerostin on osteoclastic activity. Researchers found that mechanical loading decreased the potential of osteocytes to induce osteoclast formation by direct cell-cell contact [8]. Wijenayaka et al found that sclerostin might have a catabolic action through the promotion of osteoclast formation and activity by osteocytes, in a RANKL-dependent manner [25]. Our in-vitro study showed that rhSCL increased the osteocyte expression of RANKL at the transcript and protein levels. In addition, the co-culture experiment demonstrated that rhSCL interference increased TRAP-positive cells of the osteoclast precursor cells, indicating that sclerostin improves the osteoclast induction ability of osteocytes, which is in accordance with the tooth movement experiment. Our previous published researches demonstrated that sclerostin was involved in alveolar bone loss in periodontitis and occlusal hypofunction [40, 41]. These indicated that sclerostin played an important role in alveolar bone remodeling. To further illustrate the function of sclerostin on tooth movement, local or systematic application of sclerostin proteins or sclerostin monoclonal antibodies in tooth movement should be performed in the future studies to investigate whether these agents regulate bone remodeling during tooth movement.

In conclusion, the expression of sclerostin in the compression site was maintained at a high level during the 1^st^ week of tooth movement, which was induced by a hypoxic environment and related to osteoclastogenesis at the compression site. Sclerostin deficiency decreased the osteoclastic activity in compression site during tooth movement. The function of sclerostin to promote osteoclastic activity was partially attributed to increasing RANKL expression of osteocytes.

## Supporting Information

S1 Fig IHC staining of sclerostin in the tailbone of mice. (A) No positive staining of sclerostin was determined in the SOST KO mice. (B) Highly expressed sclerostin in the WT mice. The white arrows indicate positive staining of sclerostin.
(TIF)

S2 Fig IHC staining of RANKL in the alveolar bone at the compression site at 8d. (A) The WT mice showed high expression of RANKL in the alveolar bone. The white arrow indicates the positive staining of RANKL. (B) The SOST KO mice showed low expression of RANKL in the alveolar bone.
(TIF)
