# Supplementary figures and images for: Sclerostin Promotes Bone Remodeling in the Process of Tooth Movement
Source: PLoS One. 2017 Jan 12;12(1):e0167312. doi: 10.1371/journal.pone.0167312 (PMC5230762; doi:10.1371/journal.pone.0167312)

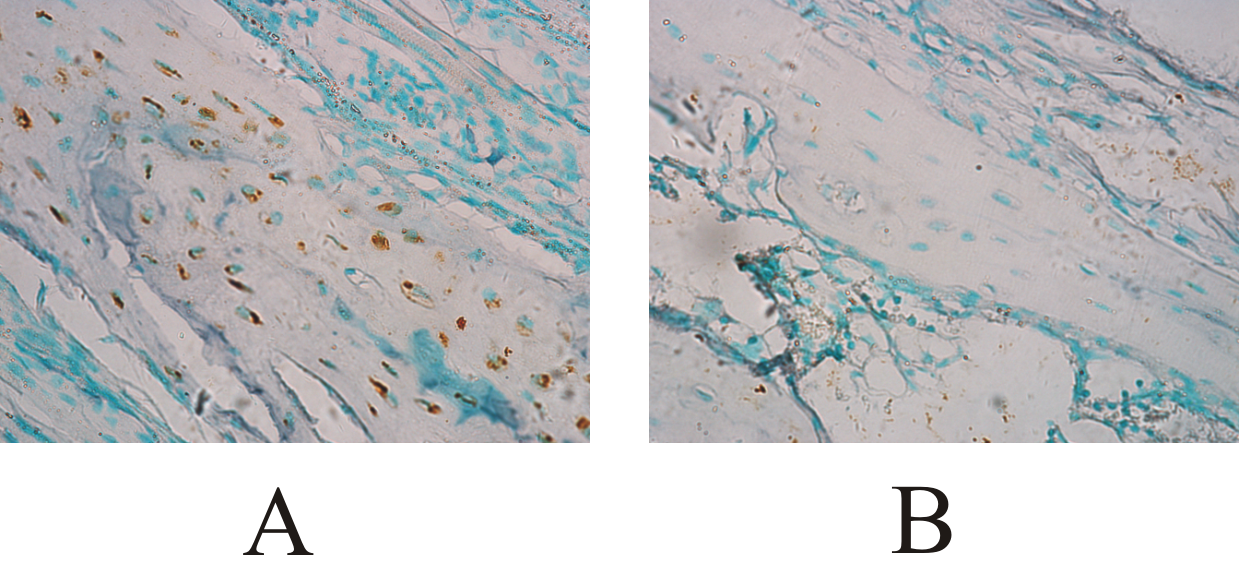

Supplement: S1 Fig — (A) No positive staining of sclerostin was determined in the SOST KO mice. (B) Highly expressed sclerostin in the WT mice. The white arrows indicate positive staining of sclerostin. (TIF) [file pone.0167312.s001.tif]

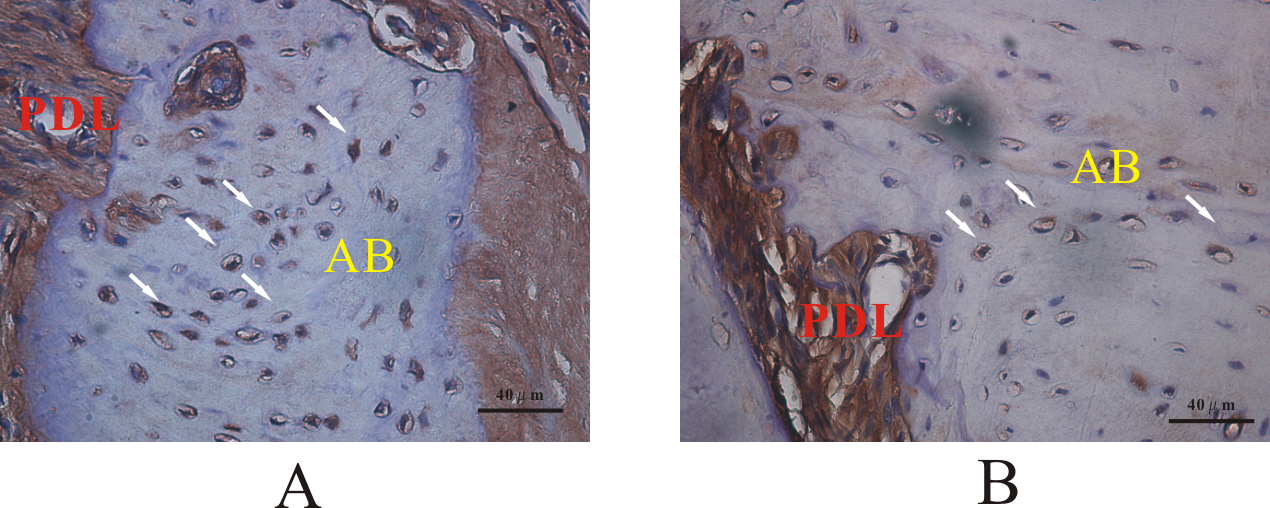

Supplement: S2 Fig — (A) The WT mice showed high expression of RANKL in the alveolar bone. The white arrow indicates the positive staining of RANKL. (B) The SOST KO mice showed low expression of RANKL in the alveolar bone. (TIF) [file pone.0167312.s002.tif]
